# BioSense: An automated sensing node for organismal and environmental biology

**DOI:** 10.1016/j.ohx.2024.e00584

**Published:** 2024-09-10

**Authors:** Andrea Contina, Eric Abelson, Brendan Allison, Brian Stokes, Kenedy F. Sanchez, Henry M. Hernandez, Anna M. Kepple, Quynhmai Tran, Isabella Kazen, Katherine A. Brown, Je’aime H. Powell, Timothy H. Keitt

**Affiliations:** a School of Integrative Biological and Chemical Sciences, The University of Texas Rio Grande Valley, Brownsville, TX 78520, USA; b Department of Integrative Biology, The University of Texas at Austin, Austin, TX 78703, USA; c Carnegie Mellon University, Pittsburgh, PA 15213, USA; d Department of Physics, The University of Texas at Austin, Austin, TX 78712, USA; e The Oden Institute for Computational Engineering and Sciences, The University of Texas at Austin, Austin, TX 78712, USA; f Cavendish Laboratory, University of Cambridge, Cambridge CB3 0HE, UK; g Texas Advanced Computing Center, The University of Texas at Austin, Austin, TX 78758, USA

**Keywords:** Bioacoustics, Remote sensing, Raspberry Pi, Avian ecology, Microclimate

## Abstract

Automated remote sensing has revolutionized the fields of wildlife ecology and environmental science. Yet, a cost-effective and flexible approach for large scale monitoring has not been fully developed, resulting in a limited collection of high-resolution data. Here, we describe BioSense, a low-cost and fully programmable automated sensing platform for applications in bioacoustics and environmental studies. Our design offers customization and flexibility to address a broad array of research goals and field conditions. Each BioSense is programmed through an integrated Raspberry Pi computer board and designed to collect and analyze avian vocalizations while simultaneously collecting temperature, humidity, and soil moisture data. We illustrate the different steps involved in manufacturing this sensor including hardware and software design and present the results of our laboratory and field testing in southwestern United States.

## Graphical abstract

**Figure:**
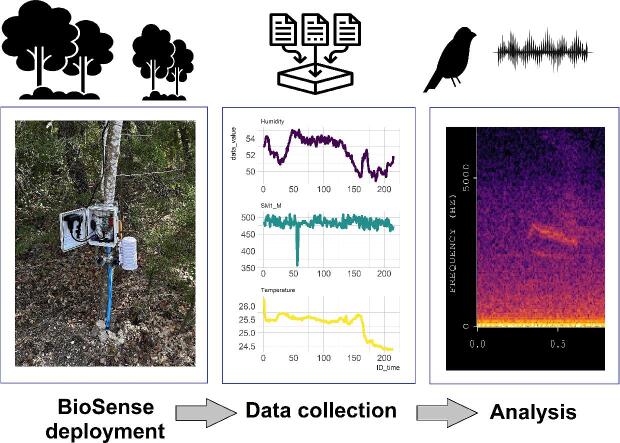

Specifications table.

**Table:** 

Hardware name	BioSense: Automated Sensing Node
Subject area	Biological sciences
Hardware type	Field measurements and sensors
Closest commercial analog	No commercial analog is available
Open source license	Creative Commons Attribution 4.0 International
Cost of hardware	$500 (USD)
Source file repository	https://doi.org/10.5281/zenodo.13345353

## 1 Hardware in context

Real-time wildlife and environmental monitoring via automated remote sensing is set to transform the field of ecology and environmental sciences [1]. A large-scale network of acoustic sensors used in conjunction with a system that collects microclimate parameters can reveal crucial biological changes, such as species composition turnover, and their interactive response with phenological patterns of the surrounding vegetation [2]. However, a cost-effective approach capable of leveraging recent technological advancements is currently lacking and hindering the collection of fine-scale and high-resolution data locally and globally [3]. While there are commercially available data loggers that can record wildlife vocalizations, weather conditions, and soil moisture data, we are not aware of any low-cost, open-source, and customizable devices currently available on the market that combine sound and microclimate sensors and, importantly, real-time analysis of bioacoustics data. Bioacoustics analyses explore the origin and frequency of sound waves and are used to make inferences about wildlife behavior and occurrence [4]. For example, long-term sound recording devices generate data that are used to detect mammal, amphibian, bird calls or insect chirps and provide insight into behavior, species composition, and movements throughout the landscape over time [5], [6], [7], [8]. However, the process of analyzing audio data and converting it into biologically meaningful soundscapes is lengthy, costly, and requires highly-trained personnel. Here, we describe the design and manufacturing process of BioSense, a relatively low-cost sensor node based on Raspberry Pi technology. BioSense operates as a waterproof and low-maintenance field unit for acoustic and environmental data acquisition, analysis, and can perform data transfer over a wireless local area network (e.g., communication hub). Each node is made from off-the-shelf components and capable of in-situ bioacoustics analysis through BirdNET, an artificial neural networks classifier for avian species detection [9]. In its current design, our sensor node monitors avian community compositions while collecting temperature, humidity, and soil moisture data at two different depths under the ground. We illustrate the different steps involved in sensor manufacturing, including hardware and software design, and present the results of our laboratory and field testing in southwestern United States.

## 2 Hardware description

Each BioSense node is a low-cost automated sensing unit for organismal and environmental biology studies. It can be programmed through an integrated Raspberry Pi computer board following the source code provided in Table 1. BioSense is designed to offer parameter customization flexibility to suit specific research questions, power constraints, and field conditions. Most of the electronic parts are housed in a Polyvinyl Chloride (PVC) box with fitted caps clamped down with stainless steel bolts and nuts. The unit is powered by a standard cable of 20 m in length, but it could also be connected to a solar panel. Bioacoustics and microclimate data can be transferred over a wireless local area network and/or saved in a local secure digital (SD) memory card. For this prototype, we used a commercially available microphone (Andrea SoundMAX) with maximum sampling input at 1 KHz of 115 dB (3 % THD at 3.7 V), maximum output (THD<3% at 1 KHz) between 24–120 mVms, sensitivity at 1 kHz ranging from −40 to −37dBV, and frequency response ranging 50–8000 Hz. The two main sensors that we integrated within the BioSense node are a STEMMA soil sensor (Adafruit Industries) and a BME280 environmental sensor (Bosch). The soil sensor has an operating voltage of 3.0–5.0 V, measuring range of 0–10 pH, and accuracy of ± 0.1 pH [10]. The BME280, a factory-calibrated (Table 2) digital sensor, provides temperature resolution of 0.01 °C, humidity maximum accuracy of 3 %, and pressure accuracy of 1 hPa [11].

**Table 1:** Design files used to configure a BioSense node.

**Design file name**	**File type**	**Open source license**	**Location of the file**
Hardware Assemble	Figures (jpeg)	CC BY 4.0	https://doi.org/10.5281/zenodo.13345353
BioSense Configuration	Config file (pdf)	CC BY 4.0	https://doi.org/10.5281/zenodo.13345353

**Table 2:** Bill of materials used to manufacture a BioSense node. Additional source links and parts numbers are included as supplementary material (S1).

**Designator**	**Component**	**Number**	**Cost per unit −currency**	**Total cost −** **currency**	**Source of materials**	**Material type**
Raspberry Pi	Raspberry Pi 4 RAM 4 GB	1	$75.00	$75.00	https://www.raspberrypi.com	Other
Sensor	Sensor BME280	1	$14.95	$14.95	https://www.adafruit.com	Other
Capacitive	Soil Moisture Sensors	2	$7.50	$15.00	https://www.adafruit.com	Other
Microphone	Andrea Stereo Microphone	1	$39.97	$39.97	Amazon	Other
Charger (optional)	50 W Voltaic Solar Charger Kit	1	$89.00	$89.00	Amazon	Other
Hardware Assemble	Qwiic PiHat	1	$9.94	$9.94	Amazon	Other
Hardware Assemble	Organic Light emitting Diodie (OLED) Screen	1	$2.99	$14.99	Amazon	Other
Hardware Assemble	Real Time Clock (RTC)	1	$6.61	$6.61	Amazon	Other
Adapter	Raspberry Pi Power Adapter with switch	1	$10.99	$10.99	Amazon	Other
Hardware Assemble	Power button	1	$0.58	$6.58	Amazon	Other
Hardware Assemble	Wi-Fi Coaxial Cable	1	$4.79	$4.79	Amazon	Other
Hardware Assemble	Wi-Fi Booster Omnidirectional Antenna	1	$4.67	$4.67	Amazon	Other
Hardware Assemble	USB Wi-Fi adapter	1	$9.99	$9.99	Amazon	Other
Hardware Assemble	Quick & Tight (QT) Connectors	2	$2.44	$4.88	Amazon	Other
Hardware Assemble	4 Wire Cable	1	$1.70	$1.70	Amazon	Other
Hardware Assemble	Japanese Solderless Terminal (JST) − Female Cable	2	$1.50	$3.00	Amazon	Other
Hardware Assemble	Quick & Tight (QT) − Female Cable	1	$0.95	$0.95	Amazon	Other
Hardware Assemble	Quick & Tight (QT) − Male Cable	2	$0.95	$1.90	Amazon	Other
Hardware Assemble	Quick & Tight (QT) − Quick & Tight (QT) Cable	3	$1.25	$3.75	Amazon	Other
Hardware Assemble	Heat Shrink Tubing Pack	1	$0.02	$0.16	Amazon	Other
Hardware Assemble	General-Purpose Input/Output (GPIO) 40 Pin Extenders	1	$1.37	$1.37	Amazon	Other
BioSense Assemble	Microphone Socks	2	$2.40	$4.80	Amazon	Non-specific
BioSense Assemble	Latex Cots	2	$0.04	$0.08	Amazon	Other
BioSense Assemble	Misc Dupont Cable Pack	1	$0.06	$0.06	Amazon	Other
BioSense Assemble	Metal Plates	3	$1.62	$4.87	Amazon	Metal
BioSense Assemble	Metal L Joins	2	$0.23	$0.45	Amazon	Metal
BioSense Assemble	Machines Screws	6	$0.23	$1.38	Amazon	Metal
BioSense Assemble	Machine Screw Nuts	6	$0.23	$1.38	Amazon	Metal
BioSense Assemble	Machine Screws	3	$0.35	$1.04	Amazon	Metal
BioSense Assemble	T-pole	1	$6.98	$6.98	Amazon	Metal
BioSense Assemble	T-pole connector	1	$9.00	$9.00	Amazon	Metal
BioSense Assemble	Medium Cable Glands	3	$0.45	$1.35	Amazon	Other
BioSense Assemble	Air vent	1	$9.70	$9.70	Amazon	Non-specific
BioSense Assemble	Conduit	1	$2.98	$2.98	Amazon	Non-specific
BioSense Assemble	Conduit Gland	1	$1.18	$1.18	Amazon	Non-specific
BioSense Assemble	Sensor Box	1	$26.99	$26.99	Amazon	Non-specific
BioSense Assemble	Radiation Shield	1	$5.00	$5.00	Amazon	Non-specific
BioSense Assemble	3M Zip Tie Adhesive Mounts	2	$0.10	$0.20	Amazon	Non-specific
BioSense Assemble	3M Dual Lock Velcro	1	$2.06	$2.06	Amazon	Non-specific
BioSense Assemble	Gorilla Silicone	1	$15.69	$15.69	Amazon	Other
Hardware Assemble	Polyvinyl Acetate Printed Circuit Board Conformal	1	$25.16	$25.16	Amazon	Other
Adapter	Conntek 30,132 IEC C8 to 1-15R	1	$8.43	$8.43	Amazon	Other
Adapter	NEMA 1-15P to IEC-720-C7	1	$5.83	$5.83	Amazon	Other

The BioSense prototype node has the following main characteristics:

• Low power consumption.
• Programmable to meet the different research needs of each user.
• Low cost at $500.00 USD.

## 3 Design files summary

The design file “Hardware Assemble” shows a list of figures and illustrations useful to understand how the main components are connected. The design file “BioSense Configuration” shows how to perform node configuration of the Raspberry Pi with a BME280 sensor, USB microphone, and two soil moisture sensors.

## 4 Bill of materials summary

### 4.1 Build instructions

Building a BioSense node requires power tools and basic Python and command line programming skills. The assemble protocol involves three main steps: 1) preparing the protective enclosure, 2) connecting the sensors to the Raspberry Pi computer board and, 3) programming the Raspberry Pi.

*Preparing the enclosure*.

We used a 220 x170 x110 mm white PVC enclosure with a hinged lid and gasket seal to protect the electronics from the elements. The preparation of the enclosure can be described in 10 steps.

1. Gather the following parts: one 220 × 170 × 110 mm enclosure, one air vent, one conduit gland, three medium cable glands, one Wi-Fi antenna, one 3-D printed radiation shield (modified from https://www.printables.com/model/235525-solar-radiation-shield under Creative Commons 4.0 International License).
2. Using the pack of two screws provided in the box and the plastic grid, use the screws to drill the grid into place.
3. Using a step drill bit and with the enclosure facing upwards and the hinges of the lid facing to the left, drill two holes on the south facing wall under the lip of the lid. Drill the left one 20.6 mm large for the conduit gland and 51 mm to the right drill a hole 17.5 mm large for the first cable gland.
4. Drill three holes on the west side of the enclosure under the hinges of the lid. To the far left, about an inch away from the corner drill a 17.5 mm hole for the second cable gland, about 51 mm to the left in the center of the wall drill a 12 mm of a hole for the air vent, and about 102 mm to the right of the air vent an inch down, drill a 14.3 mm of a hole for the Wi-Fi antenna.
5. On the east side of the enclosure, drill in the center of the wall drill the last 17.5 mm of a hole for the cable gland, to the right of the gland about 3.175 mm drill in the radiation shield using screws provided with the enclosure.
6. Once all the holes are drilled place the cable glands, vent, conduit, and Wi-Fi antenna in their respective holes ensuring they are plenty snug and do not move once fully installed.
7. Once all holes are tight with their gland use silicone sealant around the edges of the glands to make sure they are fully sealed. An additional layer of epoxy can be used to ensure protection from water infiltration.
8. Once the sealant is fully dry, not tacky, or wet, you can place the Raspberry Pi 4 on the plastic grid with the Universal Serial Bus (USB) ports facing the south wall.
9. Fit the microphone cable through the south facing wall medium gland, the BME280 sensor through the east facing wall through the medium gland, and the power adapter and soil moisture probes through the conduit gland on the south facing wall. Some power adapters may be too large for this design, adjust the conduit size accordingly.
10. Screw the Wi-Fi cable into the end of the Wi-Fi antenna with a 6 mm wrench and screw the other end of the cable into the USB to Wi-Fi cable adapter then plug the USB into a USB port on the Raspberry Pi 4.

### 4.2 Connecting the sensors to the Raspberry Pi computer board

**Fig. 1:**
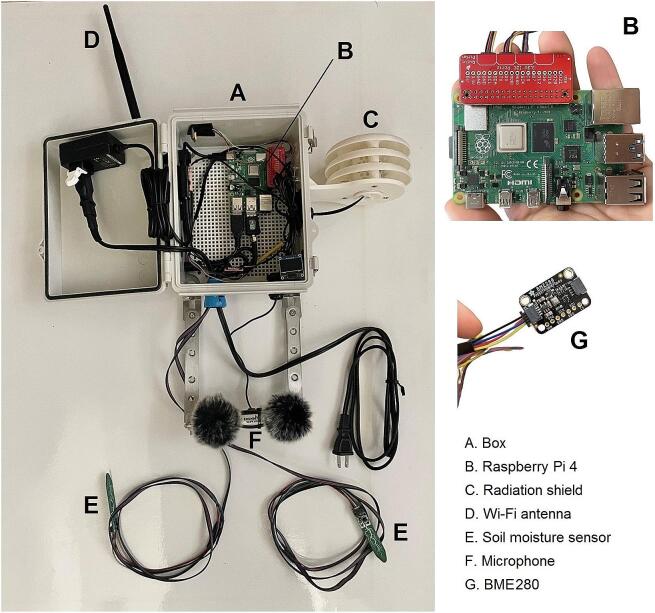
The main components of a BioSense node. The basic hardware configuration of each unit includes an integrated a Raspberry Pi computer board, a BME280 weather station for air temperature, humidity and pressure data collection protected by a radiation shield, a Polyvinyl Chloride (PVC) waterproof box fitted caps clamped down with stainless steel bolts and nuts, a stereo microphone, two soil moisture probes, and an optional solar-charged battery pack if no electricity plugs are available. A) protective enclosure, B) Raspberry Pi 4, C) radiation shield, D) Wi-Fi antenna, E) two soil moisture sensors, F) microphone, G) BME280.

**Fig. 2:**
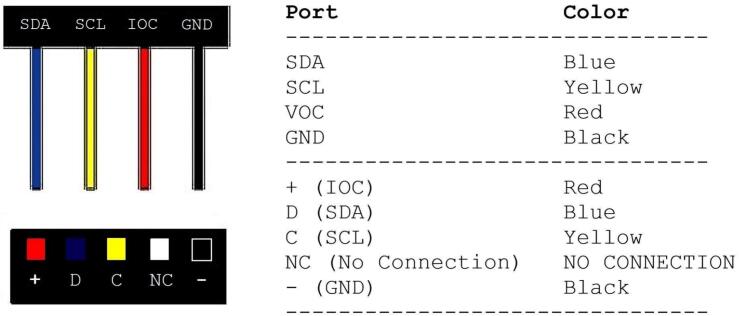
Real Time Clock (RTC) port and color table.

The BioSense’s electronic components are displayed in Fig. 1. No additional tools are required for connecting sensors. This is a beginner-level task and can be completed in less than 20 min after materials are gathered. The BME280 sensor collects real-time temperature and humidity and is protected from harsh heat and other weather conditions by the radiation shield. The soil moisture sensor setup includes two probes that can be deployed at different depths into the ground. The Real Time Clock (RTC) allows the Raspberry Pi to record the time for any region and is used when setting up cron jobs and other automated tasks (Fig. 2). Lastly, the microphone records sound data that is analyzed via BirdNet to detect avian species at the field site. Together, these elements collect four types of data, sound, soil moisture, air temperature and humidity in real time, but the analysis of audio data occurs in situ and the results can be stored in the internal SD card or transmitted via Wi-Fi, depending on the node configuration. Additional sensors may be easily added or removed as needed. The Raspberry Pi's GPIO pins are digital and for most sensors conversion from analog to digital signal is required. For rapid prototyping, users can select from a large ecosystem of digital sensors with built-in qwiic connectors, enabling plug-and-play functionality via the qwiic hat. This is how we have connected the BME280 and soil moisture sensors to BioSense. Alternatively, analog sensors may be connected to an analog-to-digital converter (ADC), which then connects to the GPIO pins either directly or via qwiic hat, as some ADCs also have qwiic connectors. With analog sensors and ADCs, one must consider the power demands of the sensor, the voltage range of its signal, and the voltage range of the ADC input pins, both to ensure safe operation and interpret the measured signal. For bioacoustic monitoring, microphones may be analog or digital. In our case, we used a microphone with a built-in analog-to-digital converter with a plug-and-play USB interface. If the user is interested in more than two audio channels, four and eight-channel microphone arrays with USB connections may also be purchased for a plug and play solution. Alternatively, users may assemble their own system of analog microphones and ADC solutions. Below, we describe the process of connecting the sensors to the Raspberry Pi (RPi) computer board in five steps.

1. Gather the following parts: one RPi, one Real Time Clock (RTC), one BME280, one mini-LCD display, one QT-QT cable, one QT-Female Dupont cable, one male Dupont cable, one Qwiic HAT for Raspberry Pi (Qwiic pHAT), one USB microphone with auxiliary connector, two finger cots, two microphone socks and 40-pin general purpose input/output (GPIO) extenders.
2. Start by placing the 40 GPIO extenders to the RPi, on top of the extenders and place the Qwiic pHAT board with the USB arrows pointing towards the USB parts on the RPi.
3. Plug the QT cables into the left side ports on the Qwiic pHAT with the QT end of the cables.
4. Plug in the other ends of the two QT-QT cables into the BME280 sensor and one into the soldered soil moisture sensors (joined together in one cable), heat shrink tubing on the cable to the BME280, the female Dupont end of the cable will connect to the LCD display, and the Male Dupont end of the cable into the RTC. Use the diagrams, photos, and Table 1 for instructions on how to plug the Dupont ends of the cable into the ports on the sensors.
5. Using the microphone USB cord, plug it into the RPi’s USB port and ensure the microphone jack (pink) is plugged into the microphone icon on the USB adapter. Once the microphone is in place, take two finger cots and slide one over each microphone. Then, put on two microphone socks on top of the finger cots. Stretch the socks lightly until they cover the entire microphone.

### 4.3 Programming the Raspberry Pi

Setting up the functionalities of the BioSense node is performed using the Raspberry Pi Integrated Development Environment. The software required for the BioSense node was designed for simplicity and immediate usability. The following libraries are used: git, pip, python3-pip, setuptools, i2c-tools, and the code repository for the BioSense platform is available along with the design file “BioSense Configuration” https://doi.org/10.5281/zenodo.13345353. We describe the Raspberry Pi programming steps below.

1. The BioSense node requires the use of a Raspberry Pi device model 4 and above, an SD card and adapter.
2. The Raspbian Operating System version that must be downloaded onto the SD card is the 64-bit Raspbian Lite. It can be installed from the Raspberry Pi Imager at https://www.raspberrypi.com.
3. Once the SD card has the OS downloaded, it must be inserted into the Raspberry Pi.
4. Ensure that the Raspberry Pi has the latest default libraries by using the 'sudo apt-get update' and 'sudo apt-get upgrade'. Interfacing with the Raspberry Pi requires a keyboard and monitor to be connected to the device via USB connection.
5. Install the git, pip, and python3-pip packages using the package manager and issue the commands 'sudo pip3 install −-upgrade setuptools' and 'sudo apt-get install −y i2c-tools'.
6. Configuring the Raspberry Pi is necessary to allow for wireless connections. The Raspberry Pi must be configured to the correct time zone for its region, must have SSH enabled, and must have I2C connections enabled.
7. If desired, the user can set up a Virtual Private Network (VPN) and a Secure Socket Shell (SSH) to connect into devices with static or dynamic Internet Protocols (IPs) for remote administration. This is achieved by creating a Zerotier account and installation using a local device, then run the commands listed in the section “Remote Administration”. Users may additionally wish to connect the device to a local network and run port scanning software, such as nmap (https://nmap.org/) from another machine on the same network. A best practice is to only enable required ports, e.g., SSH, to receive connection attempts. This can be achieved by disabling any unneeded services and enforced by only enabling the required port connections in firewall rules.
8. The setup for the bash scripts and Blinka can be completed by running the setup.bash and raspi-blinka.py files, respectively. The Fake Clock must also be disabled.
9. The Real Time Clock (RTC) can be configured by editing /boot/config.txt, and adding the line 'dtoverlay = i2c-rtc,ds3231′.
10. Crontab is necessary to launch processes of the BioSense node at designated times.
a) To continually update the display, display.py must be configured to run every minute.
b) To add a soft shutdown component, softshutdown.py is run on the Crontab @reboot event.
c) We run environmental data collection on Crontab by scheduling sensor_collect.py. Note that sensor_collect.py collects data from the BME280 and soil moisture sensors (in our github example we schedule the collection of acoustic data at sunrise and sunset for 1800 s).
11. Lastly, minio-clientsetup.bash is implemented to prepare the Raspberry Pi for the wireless transmission of files to a home server for storage or further analysis. A .env file must be created and filled with the necessary login information to access the server.

## 5 Operation instructions

### 5.1 Deployment

**Fig. 3:**
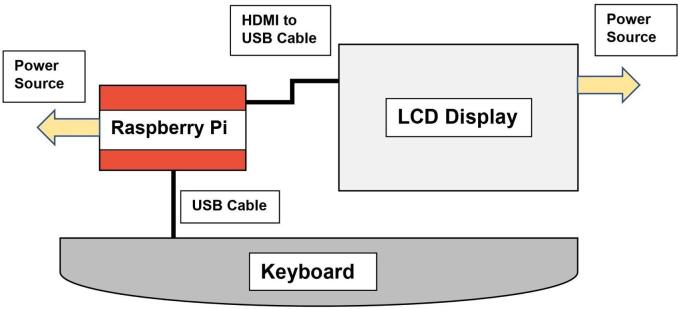
Raspberry Pi cable and power supply configuration before insertion into the waterproof case.

**Fig. 4:**
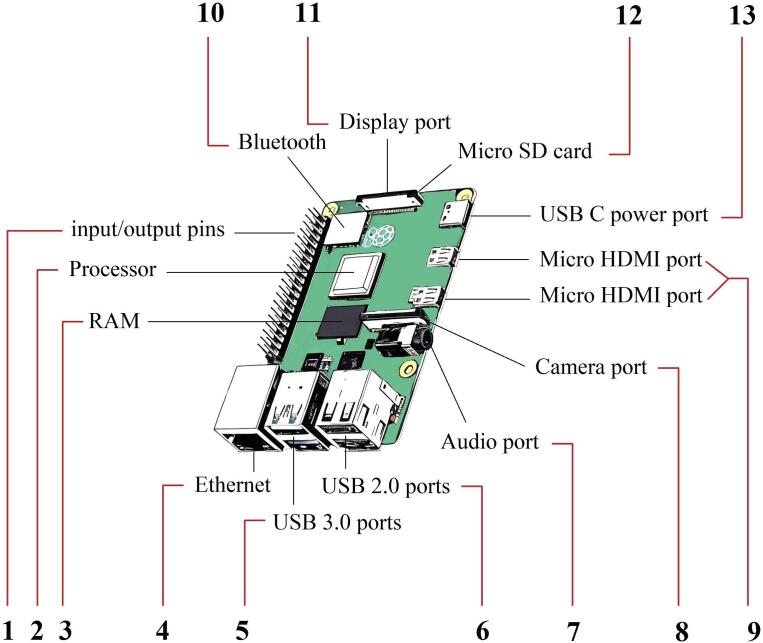
1) Set of 40 pre-soldered general purpose input/ouput (GPIO) connectors for additional sensors. 2) Quad-core Cortex-A72 (ARM v8) 64-bit and 1.5 GHz. 3) RAM up to 8 GB LPDDR4-2400 SDRAM. 4) Gigabit Ethernet. 5) Set of 2 USB 3.0 ports. 6) Set of 2 USB 2.0 ports. 7) Stereo audio port (4-pole). 8) MIPI-CSI camera port (2-lane). 9) Set of 2 Micro HDMI ports supported up to 4kp60. 10) Bluetooth 5.0, 2.4 GHz and 5.0 GHz IEEE 802.11b/g/n/ac wireless LAN. 11) MIPI-CSI display port (2-lane). 12) Micro SD card is placed under the display port. 13) USB C power port (5V/3A).

We ran several laboratory tests (e.g., indoor tests) before field deployment and we recommend testing the Raspberry Pi computer board before insertion into the waterproof case following these steps:

1. Connect the RPi to the keyboard by attaching the keyboard USB cable into a USB port on the RPi (Fig. 3; Fig. 4; Fig. 5).

**Fig. 5:**
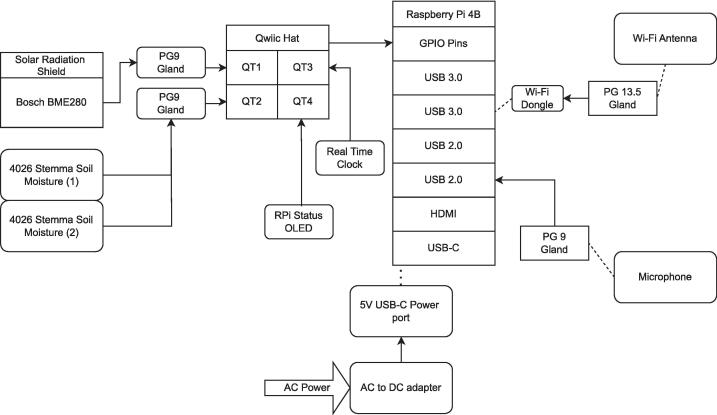
BioSense configuration workflow.
2. Install the HDMI section of the HDMI-USB cord into the LCD display’s HDMI port.
3. Install the USB end of the HDMI-USB Cord into the USB port on the RPi.
4. Plug in the power source that came with the RPi into the USB C port of the RPi and the other end into the wall power plug.
5. Install the LCD power source that came in the box into the USB-micro port and the other end into the wall power plug.

Alternatively, for a RPi setup using a desktop computer monitor, we recommend testing the RPi 4 following these steps:

1. Connect the RPi to the keyboard by attaching the keyboard USB cable to the RPi USB port.
2. Connect the HDMI Micro end of the HDMI Micro-HDMI cord into the RPi.
3. Connect the HDMI end of the HDMI Micro-HDMI cord into the desktop monitor.
4. If the monitor has VGA ports but no HDMI, Connect the HDMI end of your wire into the HDMI end of the HDMI-VGA cord (optional).
5. Connect the VGA end of the HDMI-VGA cord into the desktop monitor (optional).
6. Plug in the power source that came with the RPi into the USB C port of the RPi and the other end into the wall power plug.
7. Plug in the power source that came with the desktop monitor into its designated port and the other end into the wall power plug.

## 6 Validation and characterization

### 6.1 Data collection and analysis

**Fig. 6:**
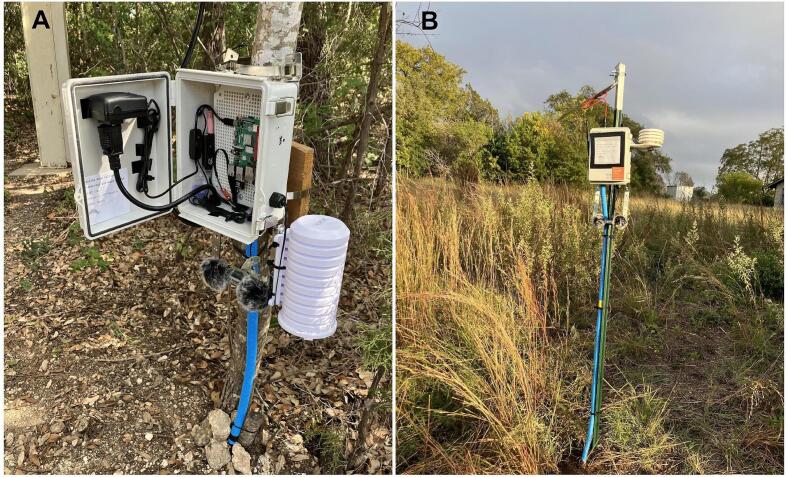
Examples of BioSense prototypes deployed in the field at the Brackenridge Field Laboratory, Texas (panel A), and at the Balcones Canyonlands Preserve, Texas (panel B). Note that the radiation shield in panel A can be modified to accommodate more than three protective discs, if needed.

**Fig. 7:**
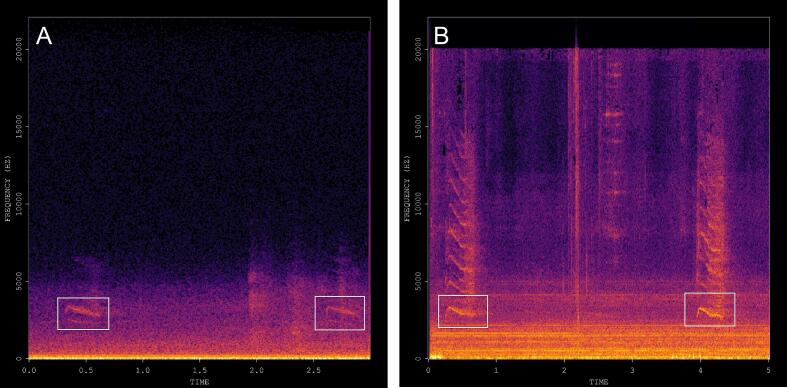
Blue Jay vocalization spectrogram extracted from the BioSense recording with the highest confidence level (0.31) as provided by BirdNET analysis (panel A). Blue Jay vocalization spectrogram obtained with a separate recording unit while visually confirming the species sighting nearby the microphone for comparison purposes (panel B). The spectrogram of the calls inside the white rectangles in panel A are nearly identical to the Blue Jay signature calls plotted inside the white rectangles in panel B. This result represents a simple validation of the BirdNET analysis implemented through a Raspberry Pi platform which we embedded into the BioSense node configuration. While a thorough investigation of avian species occurrence at the test site is beyond the scope of this manuscript, this illustration provides evidence that at least for common species such as the Blue Jay, BioSense can perform a neural network classification analysis and detect the correct species. (For interpretation of the references to color in this figure legend, the reader is referred to the web version of this article.)

**Fig. 8:**
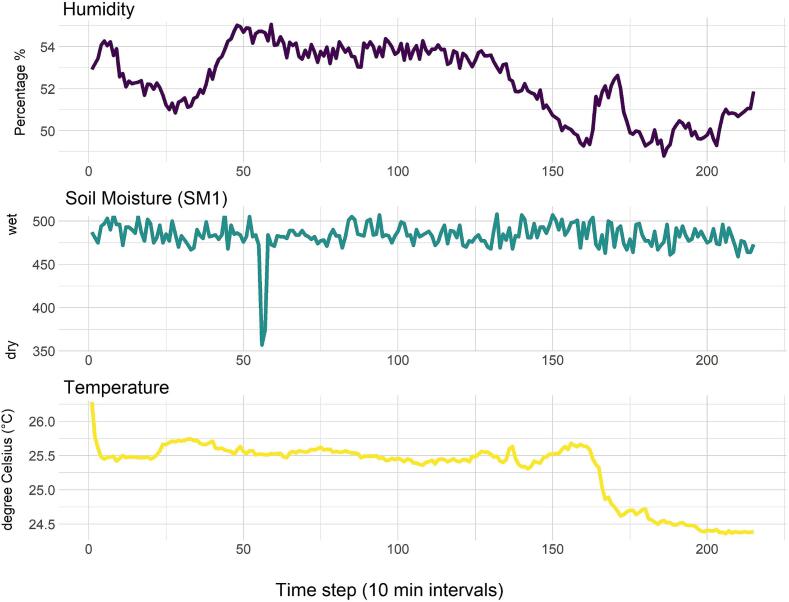
Environmental data profiles for air humidity, soil moisture, and ambient temperature during laboratory testing. The capacitive measurement through the ATSAMD10 chip in one soil moisture probe (SM1) showed a sharp decrease over two consecutive timestamps corresponding to the measurements taken on September 25 at 09:20 pm and 09:30 pm. We do not have a clear explanation for these observations, but it is possible that we may have recorded a couple of faulty capacitive readings and we recommend caution with the interpretation of the results. Potential faulty readings and data outliers could be mitigated by averaging and/or filtering multiple data points. Overall, air temperature and humidity recorded by the BME280 probe ranged between 24.3 °C and 26.2 °C and 48.8 % and 55 %, respectively. These measurements are consistent to the controlled temperature and humidity laboratory condition in which we tested our BioSense prototypes.

We deployed multiple BioSense prototypes in the field, to ensure the reliability of the weatherproof case, as well as in the laboratory at the University of Texas at Austin to test data collection capabilities. The testing field sites in Texas were located at the Brackenridge Field Laboratory, an urban research station that is part of the University of Texas at Austin, and at the Balcones Canyonlands Preserve, Texas (Fig. 6). We report the data collection results from our indoor laboratory assessment as they provided the best testing conditions for monitoring the performance of our final prototype (Table 3; Fig. 7; Fig. 8). We operated a BioSense prototype for 36 h with no interruptions while measuring indoor ambient parameters at 10-minute intervals and simultaneously recording and analyzing avian vocalizations at sunset and sunrise over two consecutive days on September 25 and September 26, 2023, at the University of Texas at Austin. Thus, we tested the dependability of our hardware and software design as well as the Raspberry Pi 4 capability to perform a neural network classification analysis of avian vocalizations [9].

**Table 3:** BirdNET results. Blue Jay and Red-tailed Hawk vocalizations were both detected six times and the Blue Jay had the highest confidence level (0.31). Both species are commonly seen at the testing sites during the fall and vocalize often. While an exhaustive species occurrence survey is beyond the scope of this research, all the other five species detected by BioSense through BirdNET analysis occur in central Texas in large numbers and can be observed in urban and suburban settings.

Begin Time(s)	End Time (s)	Low Freq (Hz)	High Freq (Hz)	Species Code	Common Name	Confidence
228	231	150	15,000	blujay	Blue Jay	0.25
291	294	150	15,000	blujay	Blue Jay	0.11
303	306	150	15,000	rethaw	Red-tailed Hawk	0.16
345	348	150	15,000	gockin	Golden-crowned Kinglet	0.12
354	357	150	15,000	gockin	Golden-crowned Kinglet	0.13
423	426	150	15,000	blujay	Blue Jay	0.19
426	429	150	15,000	rethaw	Red-tailed Hawk	0.12
429	432	150	15,000	rethaw	Red-tailed Hawk	0.2
714	717	150	15,000	blujay	Blue Jay	0.14
717	720	150	15,000	rethaw	Red-tailed Hawk	0.19
741	744	150	15,000	rethaw	Red-tailed Hawk	0.14
753	756	150	15,000	rethaw	Red-tailed Hawk	0.11
768	771	150	15,000	rethaw	Red-tailed Hawk	0.12
858	861	150	15,000	blujay	Blue Jay	0.19
861	864	150	15,000	blujay	Blue Jay	0.31
969	972	150	15,000	whbnut	White-breasted Nuthatch	0.1
1272	1275	150	15,000	brncre	Brown Creeper	0.21
1434	1437	150	15,000	mallar3	Mallard	0.11
1452	1455	150	15,000	hergul	Herring Gull	0.17
1542	1545	150	15,000	brncre	Brown Creeper	0.19

### 6.2 Discussion

Modern ecology and environmental sciences are set for significant transformation with the advent of real-time and automated monitoring through remote sensing [1], [12]. A large array of acoustic sensors for regional or local monitoring (e.g., single node) in conjunction with a microclimate parameter collection system, can discern critical biological changes affecting population composition shifts in response to phenological patterns [13]. Recent technological innovations in autonomous recording units (ARUs) for eco-acoustic monitoring are facilitating the collection of large volumes of data worldwide. However, significant challenges persist, including variations in costs, quality standards, and the lack of consistent availability of ARU electronic components due to supply chain shortages. Moreover, ARU users need to select the most cost-effective equipment relative to their research questions and discerning between a fast growing range of off-the-shelf devices as well as custom-built options. In addition to the popular AudioMoths (Open Acoustic Devices), Song Meter series (Wildlife Acoustics), and BAR-LT (Frontier Labs), several open-source acoustic recorders are becoming available, including SOLO [14], AURITA [15], and SAFE [16], [17]. For additional details and approach comparisons we refer to [18], [19], [20]. Some commercially available devices can record and use audio data to identify bird species, but may not offer broad customization options or interchangeable sensors for collection of microclimate data. For example, the Haikubox uses a proprietary “BirdNET for Haikubox*”* neural network to compute spectrograms and share bird identification outputs via a mobile phone application as well as a web interface. Likewise, the Portable Universe Codec (PUC) bioacoustics platform (BirdWeather) operates with stereo microphones and performs neural network analysis on a cloud server using the BirdNET algorithm. The PUC system includes WiFi, GPS, and environmental sensors. However, the PUC data collection and storage is not customizable beyond the range of parameters determined by the manufacturer.

Here, we designed and assembled a highly flexible device that combines sound and microclimate sensors with real-time analysis of bioacoustics data. The result is BioSense, a multiparameter data logger using off-the-shelf components and Raspberry Pi technology as a low-cost instrument for monitoring biological and environmental changes across the landscape.

Assessing daily species occurrence is critical for understanding how organisms respond to variable environmental conditions and severe weather events [21], [22], [23]. Temperature and soil moisture data are necessary to assess drought severity and frequency and recognize patterns of vegetation growth [24]. Moreover, soil moisture can be used as a predictor of the abundance of food resources available to local wildlife and migratory birds [25]. Sound-recording methods can detect bird calls and insect chirps, for example, and can offer insights on animal movements such as the onset of bird migration [26], [27]. However, analysis of large acoustic data sets often requires the use of machine learning and artificial neural networks classifiers [28]. Here, we leveraged a neural network model architecture that separates the recorded audio into three second segments and classifies hundreds of avian species [9]. Although the methodology underlying artificial neural networks classifiers is beyond the scope of BioSense development, it is worth noting that machine learning approaches in avian occurrence and behavioral studies have limitations, and further testing along with model training are necessary to improve the accuracy and reliability. Several research efforts have proposed methods to improve the BirdNET algorithm and conducted comparative analysis of automated bird sound classifications [29], [30], [31], [32], [33], [34]. In particular, research by Toenies and Rich (2021) showed that the effectiveness of BirdNET in accurately identifying avian species is dependent on the species being recorded as well as the microphone and model of the ARU being used [35]. To provide a more comprehensive assessment of BirdNET's performance across different conditions and datasets, future research should concentrate on understanding how various vocalization attributes, including duration and repertoire range, affect the system's ability to make correct classifications as well as testing high-performance microphones under extreme field conditions [36]. Nevertheless, our design offers plenty of flexibility as the full extent of the audio data can be stored in relatively large SD cards (400 GB) and subsequently retrieved for additional analysis on external computer clusters. Moreover, the BioSense node we assembled presents an open-source alternative for in situ wildlife and environmental monitoring that can be connected to the internet via Wi-Fi and create a regional sensor network.

BioSense could be further customized to include other probes to measure pH, light intensity, wind, and air pollution to satisfy a range of research objectives. The performance of the BME280 sensor under field conditions has been assessed with generally positive results [37]. However, earlier studies have highlighted the potential impact of the enclosure on accurate humidity measurements of the sensor [38]. Similarly, the audio recording analysis could be expanded to include a neural network trained on sounds from different species to perform insect, amphibian, and mammal classifications. Furthermore, in addition to the probes and microphones that we used, we acknowledge that there are several commercially available alternatives that could be connected to BioSense to measure environmental parameters (i.e., soil moisture, temperature, humidity) with higher precision and accuracy. However, alternative models might be more expensive, may present compatibility issues with the Raspberry Pi platform, and may require comparative analysis to assess performance, reproducibility, and potential design pitfalls. Yet, cost reductions should never undermine the accuracy and quality of the sensors. We emphasize the importance of maintaining a balance between reducing expenses and ensuring high standards in autonomous data collection.

Here, we demonstrated how BioSense may be applied to avian and environmental research and highlighted its novelty by combining data collection and real-time analysis as well as its flexibility in programming customization and potential for expansion into a regional network. We expect that new BioSense prototypes will be developed in the future as technological advances become available to the scientific community [39]. However, any novel technology can be added to the current basic configuration and expand the node capabilities by including additional sensors and wireless networks. To achieve deployment optimization across an extended automated bird sound classification network (e.g., regional scale), we recommend developing a centralized data repository (CDR) dedicated to the collection, analysis, and storage of data from various nodes. A functional CDR would need to be integrated with an automated pipeline for data processing and quality control assessment for collecting audio files and microclimate measurements in standardized formats. Standardization is necessary to process information received from different nodes throughout the network and ensure data comparison over time and across sites. Moreover, the implementation of a cyberinfrastructure (e.g., cloud storage) for wireless remote access would provide monitoring tools to track system performance, detect sensor malfunctions, battery level, and ensure consistent operation across the sensor network.

Integrating passive acoustic monitoring and generating species-specific soundscapes locally and regionally can establish novel baseline information useful in data integration and comprehensive environmental analysis [40], [41]. Aggregating data can ultimately contribute to understanding fundamental mechanisms in ecology, evolution, and environmental science. For example, long-term climate perturbations and severe weather events not only have a direct impact on the survival and resilience of species, but are likely to affect their communication patterns and modify soundscape dynamics [42], [43]. Therefore, monitoring changes in soundscape characteristics after major ecosystem disturbances, along with the collection of detailed environmental data from integrated autonomous sensors, can provide valuable insights for restoration practices [44]. This approach increases our understanding of how entire ecosystems react to short-term and long-term changes, and offer better guidance in conservation efforts and environmental impact assessments.

## 7 Ethics statements

This work does not involve human subjects or experiments directly on animals.

## CRediT authorship contribution statement

**Andrea Contina:** Writing – original draft, Methodology, Formal analysis, Data curation, Conceptualization. **Eric Abelson:** Writing – review & editing, Methodology, Conceptualization. **Brendan Allison:** Writing – review & editing, Supervision, Software, Formal analysis, Data curation, Conceptualization. **Brian Stokes:** Methodology. **Kenedy F. Sanchez:** Writing – review & editing, Methodology. **Henry M. Hernandez:** Writing – review & editing, Software, Methodology. **Anna M. Kepple:** Software, Methodology. **Quynhmai Tran:** Software, Methodology. **Isabella Kazen:** Methodology. **Katherine A. Brown:** Supervision, Conceptualization. **Je’aime H. Powell:** Supervision, Software, Resources, Project administration, Methodology, Conceptualization. **Timothy H. Keitt:** Writing – review & editing, Supervision, Software, Resources, Project administration, Methodology, Funding acquisition, Conceptualization.

## Declaration of competing interest

The authors declare that they have no known competing financial interests or personal relationships that could have appeared to influence the work reported in this paper.
