# Supplementary material for: BioSense: An automated sensing node for organismal and environmental biology
Source: HardwareX. 2024 Sep 10;20:e00584. doi: 10.1016/j.ohx.2024.e00584 (PMC11417332; doi:10.1016/j.ohx.2024.e00584)
Supplement: Supplementary Data 1 [file mmc1.docx]

| **Material** | **Part Number**  **(if applicable)** | **Source Link** |
| --- | --- | --- |
| Raspberry Pi 4 Model B |  | <https://www.raspberrypi.com/products/raspberry-pi-4-model-b/> |
| Bosch BME280 |  | [https://www.amazon.com/Adafruit-BMP280-Barometric-Pressure-Altitude/dp/B013W0RR6Y/ref=sr_1_2?](https://www.amazon.com/Adafruit-BMP280-Barometric-Pressure-Altitude/dp/B013W0RR6Y/ref=sr_1_2) |
| Soil Moisture Sensors |  | [https://www.amazon.com/Adafruit-4026-Stemma-Soil-Sensor/dp/B07M7PNVG7/ref=sr_1_1?](https://www.amazon.com/Adafruit-4026-Stemma-Soil-Sensor/dp/B07M7PNVG7/ref=sr_1_1) |
| Andrea Microphone |  | [https://www.amazon.com/Andrea-C1-1019800-1-ARRAY-2S-Unidirectional-Microphones/dp/B00EHX56DC/ref=sr_1_8?](https://www.amazon.com/Andrea-C1-1019800-1-ARRAY-2S-Unidirectional-Microphones/dp/B00EHX56DC/ref=sr_1_8) |
| 10 W Voltaic Solar Charger Kit |  | <https://www.amazon.com/SOLPERK-10W-Controller-Automotive-Motorcycle/dp/B0D2XNRCZN/ref=sr_1_4> |
| Qwiic PiHat |  | [https://www.amazon.com/SparkFun-Qwiic-HAT-Raspberry-Pi/dp/B078S6HZSQ/ref=sr_1_1?](https://www.amazon.com/SparkFun-Qwiic-HAT-Raspberry-Pi/dp/B078S6HZSQ/ref=sr_1_1) |
| Organic Light emitting Diode (OLED) Screen |  | <https://www.amazon.com/Dorhea-Display-3-3V-5V-Arduino-Raspberry/dp/B07FK8GB8T/ref=sr_1_2?c> |
| Real Time Clock (RTC) |  | [https://www.amazon.com/Dorhea-DS3231-Module-Memory-Raspberry/dp/B08X4H3NBR/ref=sr_1_1?](https://www.amazon.com/Dorhea-DS3231-Module-Memory-Raspberry/dp/B08X4H3NBR/ref=sr_1_1) |
| Raspberry Pi Power Adapter with switch |  | [https://www.amazon.com/VEGET-Raspberry-Switch-Type-C-Devices%EF%BC%881-Pack%EF%BC%89/dp/B07VLW8Q6T/ref=sr_1_4?](https://www.amazon.com/VEGET-Raspberry-Switch-Type-C-Devices（1-Pack）/dp/B07VLW8Q6T/ref=sr_1_4) |
| Power button |  | [https://www.amazon.com/Cylewet-Momentary-Button-Switch-CYT1078/dp/B0752RMB7Q/ref=sr_1_14?](https://www.amazon.com/Cylewet-Momentary-Button-Switch-CYT1078/dp/B0752RMB7Q/ref=sr_1_14) |
| Wi-Fi Coaxial Cable |  | [https://www.amazon.com/Coaxial-TUOLNK-Extension-Polarity-Wireless/dp/B09D3JHQNT/ref=sr_1_27?](https://www.amazon.com/Coaxial-TUOLNK-Extension-Polarity-Wireless/dp/B09D3JHQNT/ref=sr_1_27) |
| Wi-Fi Booster Omnidirectional Antenna |  | [https://www.amazon.com/Booster-Directional-Antenna-Compact-Wireless/dp/B07WQN4KJM/ref=sr_1_15?](https://www.amazon.com/Booster-Directional-Antenna-Compact-Wireless/dp/B07WQN4KJM/ref=sr_1_15) |
| USB Wi-Fi Adapter |  | [https://www.amazon.com/wireless-USB-WiFi-Adapter-PC/dp/B07P5PRK7J/ref=sr_1_3?](https://www.amazon.com/wireless-USB-WiFi-Adapter-PC/dp/B07P5PRK7J/ref=sr_1_3) |
| Quick & Tight (QT) Connectors |  | <https://www.mouser.com/ProductDetail/Adafruit/4527?qs=OlC7AqGiEDnc1uqQf6dBpA%3D%3D> |
| 4 Wire Cable |  | [https://www.amazon.com/LEADTOPS-22AWG-Electric-Conductor-Extension/dp/B07QZVBYG8/ref=sr_1_1_sspa?](https://www.amazon.com/LEADTOPS-22AWG-Electric-Conductor-Extension/dp/B07QZVBYG8/ref=sr_1_1_sspa) |
| Japanese Solderless Terminal (JST) - Female Cable |  | <https://www.adafruit.com/product/3950> |
| Quick & Tight (QT) - Female Cable | 4397 | <https://www.adafruit.com/product/4397> |
| Quick & Tight (QT) - Male Cable | 4209 | <https://www.adafruit.com/product/4209> |
| Quick & Tight (QT) Cable | 4401 | <https://www.adafruit.com/product/4401> |
| Heat Shrink Tubing Pack |  | [https://www.amazon.com/Ginsco-580-pcs-Assorted-Sleeving/dp/B01MFA3OFA/ref=sr_1_3?](https://www.amazon.com/Ginsco-580-pcs-Assorted-Sleeving/dp/B01MFA3OFA/ref=sr_1_3) |
| General Purpose Input/Output (GPIO) 40 Pin Extenders |  | [https://www.amazon.com/Female-Stacking-Header-Compatible-Raspberry/dp/B084Q4W1PW/ref=sr_1_9?](https://www.amazon.com/Female-Stacking-Header-Compatible-Raspberry/dp/B084Q4W1PW/ref=sr_1_9) |
| Microphone Socks |  | [https://www.amazon.com/Onwon-Microphone-Windscreen-Lavalier-Microphones/dp/B07JHJF8JL/ref=sr_1_12?](https://www.amazon.com/Onwon-Microphone-Windscreen-Lavalier-Microphones/dp/B07JHJF8JL/ref=sr_1_12) |
| Latex Cots |  | [https://www.amazon.com/GF-Health-3908-Finger-Non-Medical/dp/B001EUAXSE/ref=sr_1_2_mod_primary_new?](https://www.amazon.com/GF-Health-3908-Finger-Non-Medical/dp/B001EUAXSE/ref=sr_1_2_mod_primary_new) |
| Misc Dupont Cable Pack |  | [https://www.amazon.com/Elegoo-EL-CP-004-Multicolored-Breadboard-arduino/dp/B01EV70C78/ref=sr_1_1_sspa?](https://www.amazon.com/Elegoo-EL-CP-004-Multicolored-Breadboard-arduino/dp/B01EV70C78/ref=sr_1_1_sspa) |
| Metal Plates |  | <https://www.amazon.com/Sumnacon-Stainless-Straight-Connector-Furniture/dp/B07D8XG9KB/ref=sr_1_1?c> |
| Metal L Joints |  | [https://www.amazon.com/Bracket-Stainless-Fastener-Furniture-Bedframe/dp/B08QD6PF1D/ref=sr_1_2?](https://www.amazon.com/Bracket-Stainless-Fastener-Furniture-Bedframe/dp/B08QD6PF1D/ref=sr_1_2) |
| Machine Screws A | SKU 398439 | <https://www.homedepot.com/p/Everbilt-8-32-x-1-2-in-Combo-Round-Head-Stainless-Steel-Machine-Screw-6-Pack-814201/204274786> |
| Machine Screw Nuts | SKU 346177 | <https://www.homedepot.com/p/Everbilt-8-32-Zinc-Plated-Machine-Screw-Nut-12-Pack-802141/204274122> |
| Machine Screws B | SKU 528531 | <https://www.homedepot.com/p/8-32-x-2-in-Combo-Round-Head-Zinc-Plated-Machine-Screw-4-Pack-803141/204274618> |
| T-Post |  | [https://www.amazon.com/T-Post-Bracket-3-Vertical-2-Pack/dp/B01N9YLYI6/ref=asc_df_B01N9YLYI6/?](https://www.amazon.com/T-Post-Bracket-3-Vertical-2-Pack/dp/B01N9YLYI6/ref=asc_df_B01N9YLYI6/) |
|  |  |  |
| Medium Cable Glands |  | [https://www.amazon.com/AMPELE-Plastic-Waterproof-Adjustable-Gaskets/dp/B08TCF3S13/ref=sr_1_1_sspa?](https://www.amazon.com/AMPELE-Plastic-Waterproof-Adjustable-Gaskets/dp/B08TCF3S13/ref=sr_1_1_sspa) |
| Air Vent | Mouser # 563-IPV-67801-B | <https://www.mouser.com/ProductDetail/Bud-Industries/IPV-67801-B?qs=BJlw7L4Cy7%2FU1jN1zIW2jA%3D%3D> |
| Conduit | SKU 401075 | [https://www.homedepot.com/p/Carlon-1-2-in-x-25-ft-Electrical-Nonmetallic-Tubing-Conduit-Coil-Blue-12005-025/100569078#overlay](https://www.homedepot.com/p/Carlon-1-2-in-x-25-ft-Electrical-Nonmetallic-Tubing-Conduit-Coil-Blue-12005-025/100569078#overlay) |
| Conduit Gland | SKU 577766 | <https://www.homedepot.com/p/Carlon-1-2-in-ENT-Threaded-Male-Adapter-A243D-6R/100404127> |
| Sensor Box |  | [https://www.amazon.com/QILIPSU-150x150x90mm-Universal-Waterproof-Electrical/dp/B085QCT543/ref=sr_1_6?](https://www.amazon.com/QILIPSU-150x150x90mm-Universal-Waterproof-Electrical/dp/B085QCT543/ref=sr_1_6) |
| Radiation Shield |  | <https://www.printables.com/model/235525-solar-radiation-shield> |
| 3M Zip Tie Adhesive Mounts |  | [https://www.amazon.com/Adhesive-Mounts-Holders-Multi-Purpose-Anchor/dp/B08RMS5H25/ref=sr_1_1_sspa?](https://www.amazon.com/Adhesive-Mounts-Holders-Multi-Purpose-Anchor/dp/B08RMS5H25/ref=sr_1_1_sspa) |
| 3M Dual Lock Velcro |  | [https://www.amazon.com/3M-Dual-Lock-Reclosable-Fastener/dp/B007OXK330/ref=sr_1_3?](https://www.amazon.com/3M-Dual-Lock-Reclosable-Fastener/dp/B007OXK330/ref=sr_1_3) |
| Gorilla Silicone |  | [https://www.amazon.com/dp/B09X7G4V63/ref=sspa_dk_detail_0?](https://www.amazon.com/dp/B09X7G4V63/ref=sspa_dk_detail_0) |
| Polyvinyl Acetate Printed Circuit Board Conformal |  | [https://www.amazon.com/MG-Chemicals-Silicone-Conformal-Coating/dp/B085G42TGS/ref=sr_1_3?](https://www.amazon.com/MG-Chemicals-Silicone-Conformal-Coating/dp/B085G42TGS/ref=sr_1_3) |
| Conntek 30132 IEC C8 to 1-15R |  | <https://www.amazon.com/dp/B001IZ0W5Y?_encoding=UTF8&ref_=cm_sw_r_cp_ud_dp_68GW6624FQGBE9F0GGY7&th=1> |
| NEMA 1-15P to IEC-720-C7 |  | <https://www.amazon.com/dp/B005C0COWK?_encoding=UTF8&psc=1&ref_=cm_sw_r_cp_ud_dp_5VRVZBX071S17Y8CPKC1> |
